# Agenesis of Dorsal Pancreas and Solid Pseudopapillary Tumor: Ventral Pancreas Preserving Portal Vein Resection and Reconstruction Using a Peritoneal Graft

**DOI:** 10.7759/cureus.40916

**Published:** 2023-06-25

**Authors:** Naveena AN Kumar, Arika S DSouza, Nawaz Usman, Arvind K Bishnoi

**Affiliations:** 1 Surgical Oncology, Kasturba Medical College, Manipal, Udupi, IND; 2 Cardiothoracic Surgery, Kasturba Medical College, Manipal, Udupi, IND

**Keywords:** subtotal pancreatectomy, peritoneal graft, portal vein resection, solid pseudo-papillary tumor of the pancreas, agenesis of dorsal pancreas

## Abstract

A diabetic lady in her 40s was referred to surgical oncologists with epigastric pain associated with vomiting. Computed Tomography (CT) Abdomen with contrast demonstrated a mass arising from the head of the pancreas with the absence of dorsal pancreas, confirmed on magnetic resonance cholangio-pancreatography (MRCP). A core needle biopsy was done, and the tumor was revealed to be a solid pseudopapillary epithelial neoplasm.

She underwent sub-total pancreatectomy preserving the duodenum and ventral pancreas as there was adequate free margin; however due to the tumor abutting the anterior wall of the portal vein, it was resected, and reconstruction was done using a peritoneal graft. The patient made a good recovery without any significant post-operative events.

## Introduction

Agenesis of the dorsal pancreas is a rare congenital malformation and its association with solid pseudopapillary epithelial neoplasms (SPEN) is extremely rare [1,2]. Surgery is the mainstay of treatment and if feasible, performing limited pancreatectomy is very important to avoid metabolic and endocrine complications [3]. However, SPEN involving major vessels require vascular resection and reconstruction to achieve R0 resection [4]. A variety of substitutes for vascular reconstruction have been described. The peritoneal graft is a recent concept and is not used by many [5].

We report a female patient of 47 years of age, a diabetic, with agenesis of the dorsal pancreas, diagnosed to have a SPEN arising from the head of the pancreas involving the anterior wall of the portal vein, who underwent duodenum and ventral pancreas preserving subtotal pancreatectomy with type 2 portal vein resection and reconstruction using a peritoneal graft.

## Case presentation

Clinical presentation

A 47-year-old female patient with a history of diabetes managed with oral hypoglycemic agents (T. glibenclamide 5 mg thrice daily and T. metformin 500 mg thrice daily), had presented with dull aching epigastric pain of two months duration which radiates to the back. It was intermittent which progressed to being continuous and was associated with nausea and vomiting of non-bilious nature. She had similar complaints before, and an ultrasound was done which showed features suggestive of chronic pancreatitis; therefore, she was managed conservatively. On examination, vitals were stable. General physical examination was unremarkable. Abdominal examination revealed tenderness in the epigastric region.

Investigations

At the time of referral to surgical oncologists, endosonography was done which revealed a hypoechoic lesion in the head of pancreas measuring 24x22mm with calcification abutting the superior mesenteric vein but the body and tail could not be visualized. Endoscopic ultrasound-guided fine needle aspiration cytology (FNAC) was done which revealed features suggestive of pancreatic epithelial neoplasm. 

**Figure 1:**
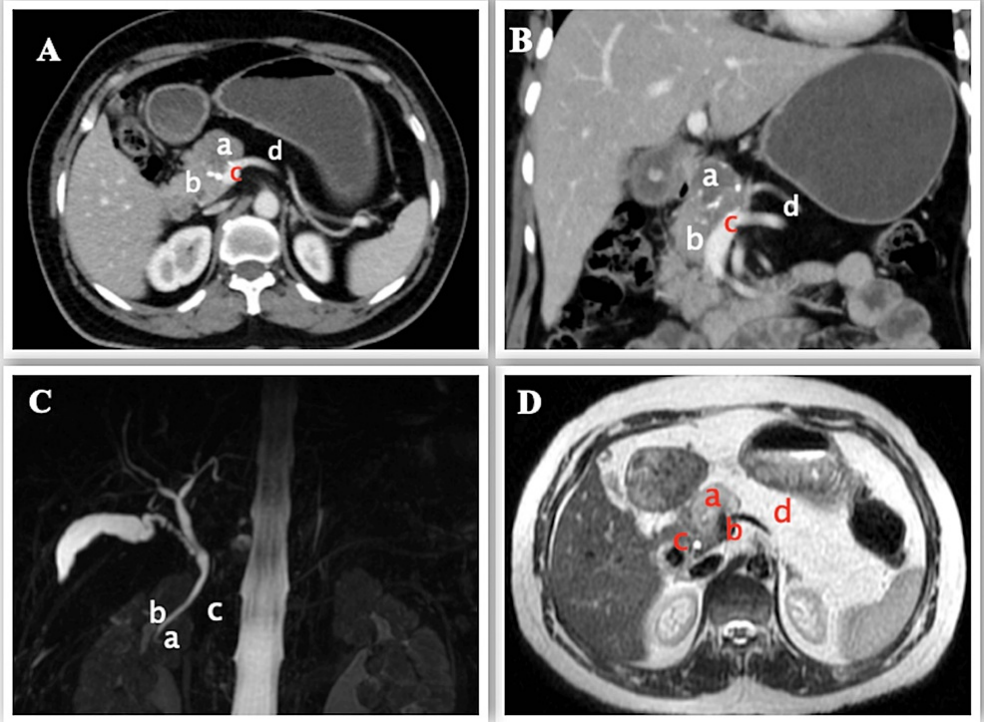
Preoperative imaging A, B - CECT (axial and coronal view) scan showing SPEN (a) arising from the head of pancreas (b) with infiltration of the portal vein (c) and agenesis of dorsal pancreas (d) C - MRCP showing normal ventral pancreatic duct (of Wirsung) (a), the common bile duct (b), and absence of dorsal pancreatic duct (c) D - MRI showing tumor (a) just above the portal confluence (b), close by distal CBD (c) and distal pancreas replaced by fat (d) CECT: contrast-enhanced computed tomography, SPEN: solid pseudopapillary epithelial neoplasm, MRCP: magnetic resonance cholangio-pancreatography, CBD: common bile duct

Following this, a triphasic contrast-enhanced computed tomography (CECT) scan showed a tumor arising from the head of pancreas with 180-degree abutment of the anterior wall of the portal vein with loss of fat planes (Figure 1A, 1B). The tumor was adherent to the common hepatic artery and gastroduodenal artery with no metastasis. However, the body and tail of the pancreas could not be visualized anterior to portal confluence and splenic vein (Figure 1A). 

On MRCP, the dorsal pancreatic duct (of Santorini) and the minor duodenal papilla could not be visualized. However, the ventral pancreatic duct (of Wirsung) and the common bile duct (CBD) were normal (Figure 1C). The distal CBD was close to the tumor (Figure 1D), with evidence of loss of fat planes with the portal vein but no evidence of loss of flow void. 

The serum amylase and lipase and tumor markers (carcinoembryonic antigen [CEA], cancer antigen 19-9 [CA 19-9]) were normal. A core needle biopsy from the tumor was reported as a solid pseudopapillary epithelial neoplasm. Our final diagnosis was agenesis of the dorsal pancreas associated with solid pseudopapillary epithelial neoplasm.

Treatment

Her case was reviewed in a multi-disciplinary team meeting with other oncological departments and opinion from vascular surgeons was sought. As the tumor was arising from the head of pancreas without dorsal pancreas, she was planned for total pancreatectomy, portal vein resection and reconstruction, and possible hepatic artery segmental resection/reconstruction to achieve R0 resection.

**Figure 2:**
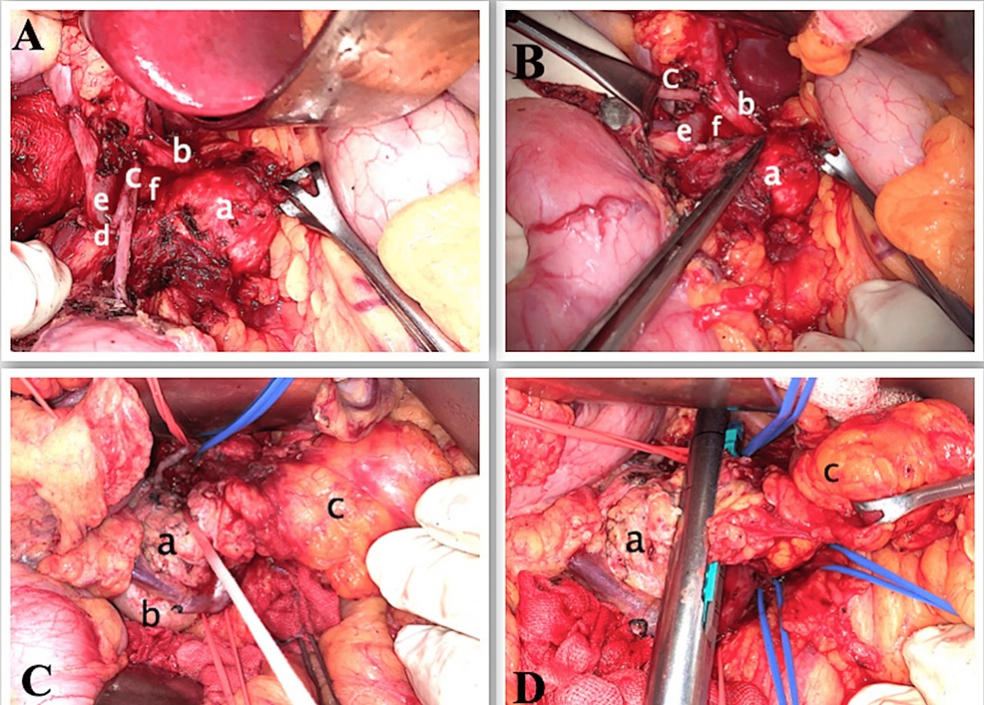
Intraoperative photos A, B - Tumor (a) freed from common hepatic artery (b) and gastroduodenal artery (c), posterior superior pancreaticoduodenal artery (clipped) (d), CBD (e), portal vein (f) C, D - Showing looping and transection of the head of the pancreas (a), duodenum (b), dorsal pancreas replaced by fat (c) CBD: common bile duct

On laparotomy through chevron incision, there was a hard tumor occupying the anterior part of the head of pancreas and the rest of the pancreas was replaced by fat with no peritoneal metastasis. As our initial plan was to perform a total pancreatectomy, the first step of surgery was to preserve the right gastroepiploic vein to maintain the venous circulation of the stomach if the splenic vein required ligation. The right gastroepiploic vein was dissected and freed along the lateral border of antro-pylorus, head of the pancreas up to the origin from the superior mesenteric vein (SMV) after ligating the gastro-colic trunk. A Kocher manoeuvre was performed. SMV was identified, looped, and traced up to the portal vein. Dissection of the common hepatic artery was commenced from the origin and the artery was looped. Meticulous dissection continued along the common hepatic artery and the artery was separated from the tumor. The gastroduodenal artery, which was adherent to the tumor was separated by meticulous dissection and taped (Figure 2A, 2B). Supra-pancreatic part of the portal vein was dissected and looped. Our plan of preserving the left gastric vein was not successful as the vein was adherent to the tumor. 

The posterior superior pancreaticoduodenal artery was sacrificed to expose the head of pancreas and the distal/intrapancreatic part of CBD (Figure 2A). The distal part of CBD was dissected and looped. Dissection along CBD was continued till the intrapancreatic part to get sufficient tumor-free margin and to safeguard the CBD. The splenic artery and vein were dissected and looped. The inferior mesenteric vein (IMV), which was draining into SMV, was dissected and looped. With the above vascular control, the posterior surface of the head of pancreas right lateral to the portal vein was dissected. As we were able to loop the head of pancreas between the tumor and intrapancreatic CBD and there was sufficient tumor-free margin at the head of pancreas, we decided to perform a duodenum and ventral pancreas preserving subtotal pancreatectomy (Figure 2C). A linear stapler was used to transect the pancreas (Figure 2D).

**Figure 3:**
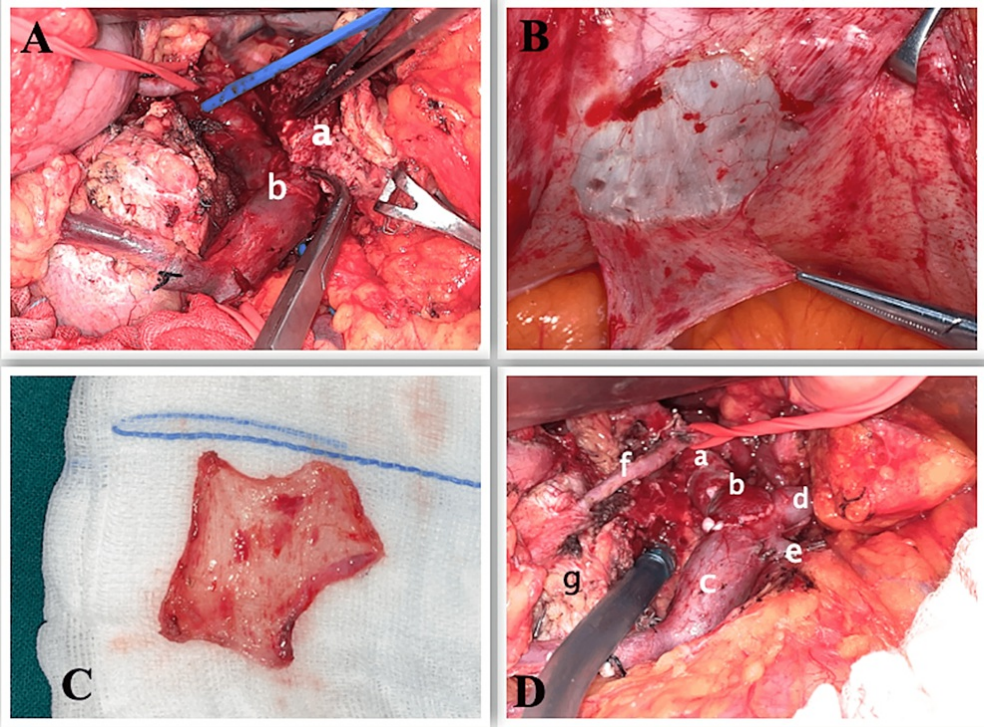
Intraoperative photos A - Tumor (a) infiltrating the portal vein (b) B - Parietal peritoneal graft harvesting from right hypochondrium C - Harvested peritoneal graft D - Portal vein (a) reconstruction with peritoneal graft (b), superior mesenteric vein (c), splenic vein (d), inferior mesenteric vein (e), gastroduodenal artery (f) supplying ventral pancreas (g)

Once the transection of the pancreas was completed, the tumor was freed from the adjacent structures except for the infiltrated part of the portal vein just above the porto-splenic junction (Figure 3A). All venous control namely, portal vein (proximally), SMV (distally), IMV, splenic vein, and right gastroepiploic vein were clamped after giving heparin infusion. Resection of a part of the wall of the portal vein was performed with sufficient tumor-free margin resulting in a defect of 1.5x1 cm on the anterolateral wall. A 2.5x2 cm sized parietal peritoneum with a thin muscular layer was harvested through the same incision from the hypochondrium (Figure 3B, 3C). The peritoneal graft was sutured to the portal vein using a continuous 6-0 polypropylene suture, making sure that the peritoneal layer was placed towards the luminal side of the vein (Figure 3D). After checking the integrity of anastomosis, all venous clamps were released. The duration of clamping was 20 minutes. The venous reconstruction was covered by the falciform ligament. The pancreatic stump was covered by omentum after placing a flat drain.

**Figure 4:**
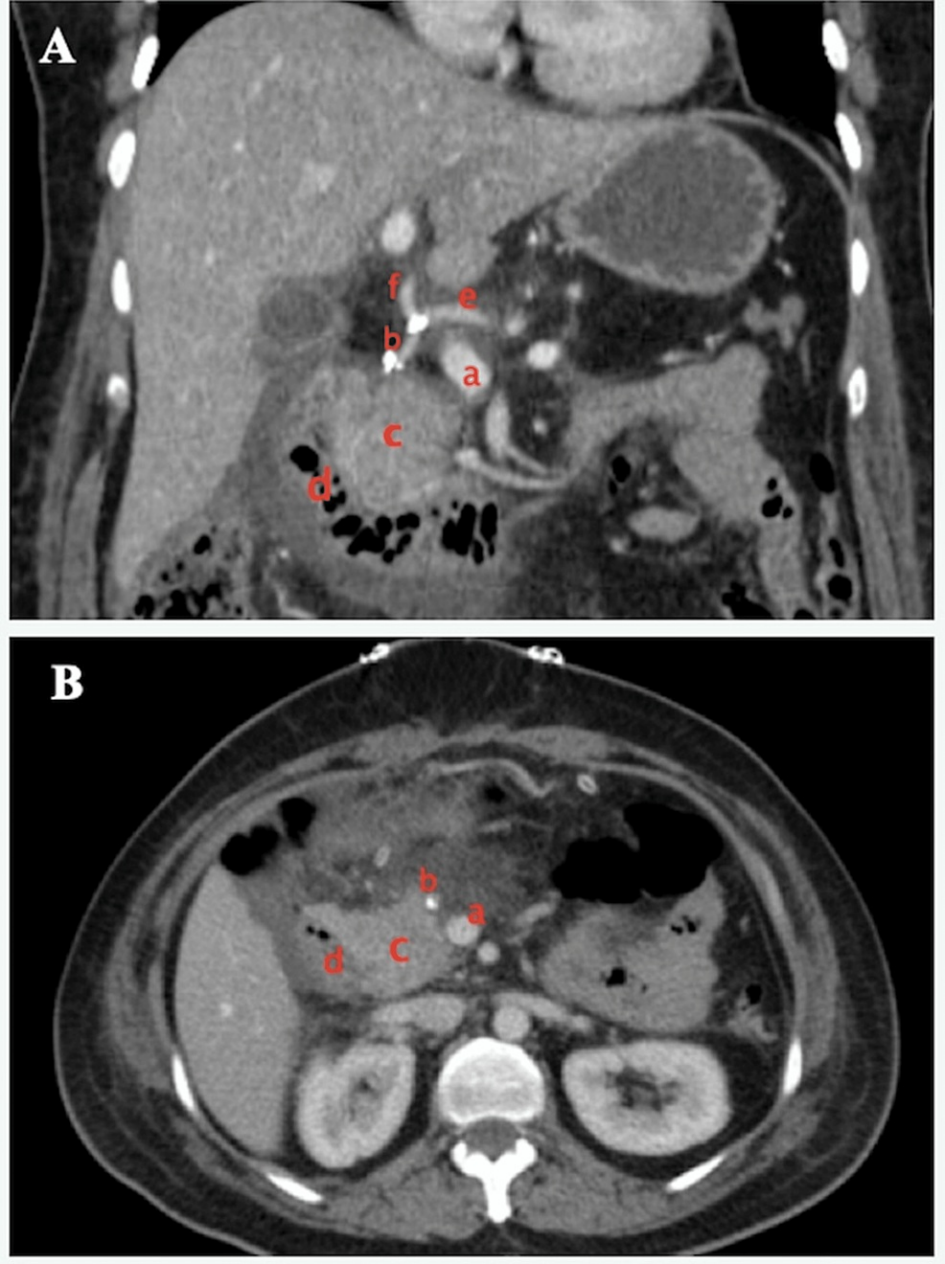
Postoperative imaging A, B - CECT scan (coronal and axial view) showing patent portal vein (a), gastroduodenal artery (b), ventral pancreas (c), duodenum (d), common hepatic artery (e), hepatic artery proper (f). CECT: contrast-enhanced computed tomography

No therapeutic anticoagulation was used except for a prophylactic dose of enoxaparin sodium given for five days as per our routine local postoperative practice. The post-operative recovery was uneventful, and the patient was discharged on postoperative day 5 (Figure 4A, 4B). The final histopathology report was SPEN with a microscopic negative margin at the transection and the presence of lymphovascular invasion and perineural invasion.

Outcome

At one month after the surgery, she came for a follow-up with no complications. At 18 months of follow-up, she was noted to be doing well with no gastrointestinal complaints. Abdominal scans were done at each follow-up to ensure venous patency.

## Discussion

The significance of this article is reporting on agenesis of the dorsal pancreas and associated SPEN, a description of the technique of duodenum and ventral pancreas preserving subtotal pancreatectomy with portal vein resection, and a detailed technique on peritoneal graft for portal vein reconstruction.

Agenesis of the dorsal pancreas is a rare congenital malformation, where the dorsal pancreatic bud fails to form the body and tail of pancreas [1,2]. It can be complete, where the minor papilla, the duct of Santorini, pancreatic neck, body, and tail are absent, or can be partial agenesis, where the tail of the pancreas is absent [1,2]. The pathogenesis of this condition is not clearly understood [1,2]. It can be asymptomatic and detected incidentally or may be symptomatic with diabetes, pancreatitis, abdominal pain, jaundice, and weight loss [1,2,6]. Associated anomalies like poly-splenia, polycystic kidney disease, congenital choledochal cysts, biliary atresia, and Kartagener syndrome have been reported [1,2]. There have been reports on the association with ampullary tumors, cystic adenocarcinoma, solid pseudopapillary tumors, intraductal papillary mucinous neoplasms, neuroendocrine tumors, and cholangiocarcinoma [2,7,8]. 

SPEN of the pancreas is rare and considered to be a tumor with a low malignant potential [9]. However, due to locally aggressive behavior, involvement of adjacent organs/vascular structures is not uncommon [9]. Surgery is the mainstay of treatment and varies from local excision and pancreatic preserving surgery to radical resection depending on the local extent [4]. It is important to perform pancreatic preserving surgery if feasible to avoid metabolic and endocrine complications [3]. 

Association of agenesis of the dorsal pancreas with SPEN is extremely rare [1]. A triphasic CECT and MRCP help confirm the diagnosis. The first case was reported in 2001 in which there was partial agenesis of the dorsal pancreas as the pancreatic accessory duct was identified and SPEN was not invading any vascular structures, hence partial resection of the pancreas was performed [10]. In 2005, the second case was reported; there was complete agenesis of the dorsal pancreas and Whipple's procedure was done to remove SPEN as the second part of the duodenum was involved. The patient was diagnosed with non-insulin-dependent diabetes mellitus before presentation, and she developed insulin-dependent diabetes in the postoperative period [11]. 

A retrospective cohort study was published in 2022 which compared the outcomes of pancreatic head-sparing enucleation for patients with solid pseudopapillary tumors associated with agenesis of the dorsal pancreas with pancreatic resections for patients with SPEN without agenesis of dorsal pancreas (ADP) in Taipei. There were three patients in the first (study) group and 25 patients in the second (control) group. There were no significant differences between the two groups in terms of pre-operative characteristics, operative measures, surgical morbidity, and post-operative complications. No lymphovascular and perineural invasion was noted in the three cases of SPEN with ADP. However, there are limitations to this study. First, the small sample size and selection bias in both groups. Second, the biases arising from the presence of different options for pancreatic resection for different locations of the tumor in the control group were inevitable. Third, a patient in the study group underwent R1 resection, for reasons not specified [12].

In a systematic review of sub-total pancreatectomy; parenchyma-sparing, limited head resections can be considered for benign or low-grade tumors [13,14]. A precise understanding of surgical anatomy, embryological development, and a proper surgical plan is very important to perform pancreas-preserving surgery. The preservation of the superior and inferior pancreaticoduodenal artery to maintain blood supply to distal CBD, duodenum, and remaining head of pancreas and the meticulous dissection of distal CBD till intrapancreatic part is the important part of the surgery. In this case, the posterior superior pancreaticoduodenal artery was sacrificed to get adequate exposure to distal CBD. We preserved the anterior superior pancreaticoduodenal artery and inferior pancreaticoduodenal artery. These procedures are more time-consuming and technically demanding [14].

As achieving R0 resection is paramount to improve long-term outcomes in a case of locally aggressive SPEN, it is necessary to perform vascular resection or multi-visceral resection if required [4]. Although most portal vein reconstructions following pancreatic surgery can be achieved by primary repair or end-to-end anastomosis, a lateral or segmental graft may be needed in 8-12% of cases [5,15]. A variety of substitutes for vascular reconstruction have been described such as autologous veins, polytetrafluoroethylene (PTFE) grafts, and cryopreserved veins [5,16]. The peritoneal graft is a recent concept and is not used by many. In a series of 30 patients, where the peritoneal graft was used for vascular reconstruction, Dokmak et al. demonstrated the safety of this procedure [5]. A parietal peritoneum with a thin muscular layer harvested from lateral hypochondrium, diaphragm, falciform ligament, and the prerenal area is ideal for portal vein repair, and thick parietal peritoneum with aponeurotic layer from the right or left rectus abdominis muscle may be suitable for reconstruction of vena cava [5]. This graft can be used as a patch graft for lateral wall defects or as a tube substitute for segmental vein reconstruction [5,17]. 

A systematic review conducted by Lapergola et al. concluded that the peritoneal graft is viable, safe, inexpensive, versatile, easily adaptable to vascular defects, not requiring extra operating time, and is a rapidly available option for vascular reconstruction during abdominal surgeries. It can be used for unplanned lateral wall vascular reconstruction, and it would reduce the reluctance of surgeons to perform vascular reconstruction when oncologically deemed [5,16]. This graft is safe even in patients with postoperative pancreatic leaks [5]. The lower thrombogenic risk may be due to the intact mesothelium and reconstruction without tension and no prolonged therapeutic anticoagulation is required [5]. With the advantage of being easily available, the simple technique of harvesting, and no reported graft-related major complications, peritoneal graft plays a major role in vascular reconstruction in the future [15].

## Conclusions

Agenesis of the dorsal pancreas is an extremely rare congenital malformation. Its association with solid pseudopapillary epithelial neoplasm is extremely rare. Sub-total pancreatectomy with preservation of duodenum and ventral pancreas should be considered in cases wherein sufficient tumor-free margins can be achieved, with a precise understanding of surgical anatomy and a proper surgical plan. This surgery could prevent the complication of pancreatic insufficiency.

Peritoneal grafts should be considered as an alternative for vascular reconstruction, especially in operations for abdominal malignancies abutting or invading vascular structures. These are versatile grafts, that can be harvested easily without any further incision and can be used for portal vein reconstruction without any major complications.
